# Efficiency of Herkogamy in *Narcissus bulbocodium* (Amaryllidaceae)

**DOI:** 10.3390/plants10040648

**Published:** 2021-03-29

**Authors:** Luis Navarro, Garbiñe Ayensa, José María Sánchez

**Affiliations:** Departamento de Biología Vegetal, Facultad de Biología, Campus As Lagoas-Marcosende, Universidad de Vigo, E-36200 Vigo, Spain; garbiayensa@hotmail.com (G.A.); jmsbot@uvigo.es (J.M.S.)

**Keywords:** hoop-petticoat daffodil, phenotypic integration, pollination biology, pollination efficiency, reproductive biology, self-interference

## Abstract

Within the theoretical framework of the correlation pleiades, floral phenotypic integration has been proposed as a consequence of selection mediated by pollinators acting on floral characters. Here, we analyzed that assumption by studying the floral biology and pollination of the late-winter species *Narcissus bulbocodium* L. We found that the flowers of *N. bulbocodium* are pollinator-dependent (mainly on *Bombus terrestris*) in terms of achieving optimal levels of seed production (xenogamy mean seed-to-ovule ratio 64%). Flowers are phenotypically integrated, and only the inclusion of the stigma within the corona seems to have a positive and significant influence on the deposition of the pollen. It has been hypothesized that by including the stigma within the corolla, the flower has some control over the contact between stigma and pollinators that could lead to an “ordered herkogamy” as a way to promote outcross and avoid self-interference. Therefore, herkogamy was also studied, and while most previous studies have assessed the evolutionary significance of herkogamy by considering its relationship with outcrossing rates, we approach this phenomenon from a novel direction assessing the relationship between a proxy for herkogamy and the precision of the pollination process. Our results seem to support the existence of an optimal herkogamy distance that could maintain maximum levels of both pollen export and (cross) pollen capture. On the basis of the broad variability of herkogamy that we have found in *N. bulbocodium* and other data in the literature, we discuss the universality of the adaptive origin of herkogamy.

## 1. Introduction

It is widely accepted that floral characters in animal-pollinated plants are under adaptation to maximize the efficiency of each pollinator visit [1]. Thus, although a strict adaptive origin has been questioned for some current plant–pollinator interactions (some of them “exaptations” rather than actual adaptations, see [1,2]) there are few doubts regarding the adaptive maintenance and tuning of those floral traits directly involved in reproduction for their potential impact on fitness [3].

The correlation pleiades of Berg [4]—broadly, the decoupling of floral from vegetative variation in animal-pollinated species, forming two distinct *correlation pleiades*—provides a theoretical framework that allows for the testing of the validity of the adaptationist arguments. In a further step, Armbruster et al. [5] have pointed out seven predictions that should be fulfilled in light of Berg’s hypothesis. The most important are (1) that those floral traits more involved in reproduction (i.e., more important for fitness) should covary (phenotypic integration), and (2) that the variation of floral traits involved in reproduction should be small, as many of them are under stabilizing selection (e.g., [6,7]).

In hermaphroditic animal-pollinated plants, floral traits optimized for one gender may be inappropriate for the other; therefore, differential selection on paternal and maternal traits may require adaptive trade-offs [1,8,9]. Herkogamy, the spatial separation of stigmas and anthers within the same flower, is considered a result of that trade-off, with the evident adaptive significance of promoting outcrossing [10] and/or avoiding self-interference [11,12]. We should therefore expect an anther–stigma separation wide enough to avoid self-pollination, but not so large to prevent the contact of pollinators with one of the sexual organs, which would reduce one of the sexual functions [9]. Considered in this way, the precision of the sexual organs positioning is key to achieve any evolutionary insight [13]. A hypothetical optimal situation could be expected at a distance such that outcrossing is maximized while self-interference is minimized [14]. Most evidence in the literature is focused on female function only and shows that there is a positive correlation between herkogamy and outcrossing (e.g., [15,16]).

*Narcissus bulbocodium* L. is a herkogamous species that may be a model to test the importance of herkogamy as a trait selected for optimizing the reproduction of flowering plants. In this study, we address this relationship between herkogamy and reproduction using a new approach. Most previous studies have found that the proportion of outcrossing pollination increases with herkogamy, even though the total number of effective pollination episodes decrease when herkogamy becomes too wide (e.g., [14]). Our approach is focused not on outcross rate, but on the relationship between herkogamy and precision, that is, the proportion of pollen that successfully reaches the stigma compared with other non-receptive floral structures. To this end, we consider the distance between the population mean anther height (pollen source) and each flower stigma height (pollen target) as a proxy for herkogamy of each flower. This approach allows us to integrate both factors—the amount of pollen reaching the flowers, and how much of that pollen is efficiently placed on stigmas—and to relate those factors with the herkogamy proxy. By studying the floral characteristics in *N. bulbocodium*, we tested the hypothesis of the universality of the adaptive origin of herkogamy, trying to answer the following questions: (1) How well “integrated” is the phenotype of flowers of *N. bulbocodium*? (2) Is there any relationship between herkogamy and mating systems, or reproductive assurance? and (3) Is there an optimal herkogamy distance, taking into account both outcross rate and precision? To fulfill those goals, we provide new data on the *N. bulbocodium* floral biology.

### 1.1. Plant Species

**Figure 1:**
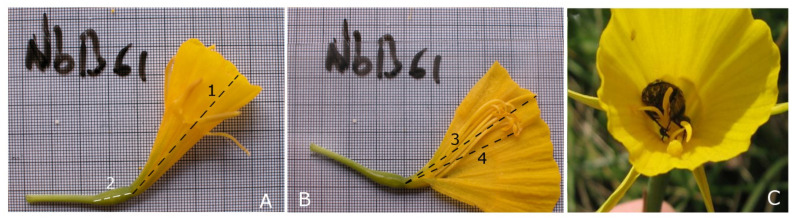
Three aspects of the flower of *Narcissus bulbocodium*: (**A**) lateral view of a flower (1: corolla length, 2: ovary length); (**B**) dissected flower exposing the sexual organs (3: style length, 4: stamen length; all stamens were measured; background in (**A**,**B**) is 1 mm graph paper); (**C**) a visitor (*Oxythyrea funesta*) at the bottom inside the corolla. Note the low fit between this pollinator and flower structure.

The hoop-petticoat daffodil *Narcissus bulbocodium* (Amaryllidaceae) is a perennial bee-pollinated bulbous herb. This large-flowered daffodil (corolla length ranging between 25 and 45 mm) commonly produces a single flower per plant. Flowers are bright yellow (Figure 1), and the corona (the innermost fused part of the perianth) is cone-shaped (identification and nomenclature after [17]).

In the northwest of the Iberian Peninsula where this study was conducted, *N. bulbocodium* is represented by scattered populations confined to poorly drained pastures. Flowering occurs from late February to mid-April, a period characterized by cool, rainy weather that frequently limits the activity of the main pollinators in the area.

### 1.2. Study Area

The present study was conducted in two random *N. bulbocodium* populations, Ortoño (42°51′10.6″ N 8°39′15.6″ W, 32 m a.s.l.) and Bertamiráns (42°51′50.1″ N 8°39′2.1″ W, 42 m a.s.l.), located in two meadows in the municipality of Ames (A Coruña province, Spain). Flowering occurs mainly in February when the typical mean temperature in the area is around 8.3 °C and total precipitation is around 167 mm, but the climate was slightly colder (mean temperature 5.1 °C) and dry (29 mm) in February 2005 when the experiments were conducted [18].

## 2. Results

### 2.1. Floral Morphology and Female Fitness

Floral characters. *N. bulbocodium* has long-living flowers, as expected for a species flowering from late winter to early spring. Mean floral lifespan was 15.4 ± 3.5 days (*n* = 25 flowers). Observation of flower maturation indicated that flowers are likely adichogamous. Anther dehiscence occurred after flower opening, and stigmas showed enzymatic activity throughout floral lifespan.

The descriptive statistics for morphometric variables are shown in Table 1. Among the measured variables, the most unpredictable character was ovary length, and the least variable was style length (coefficient of variation (CV) 21.1% and 8.7%, respectively).

**Figure 2:**
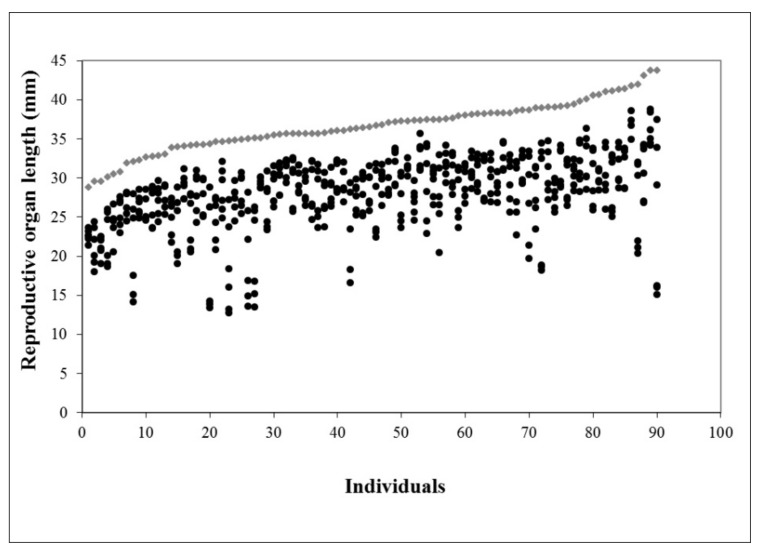
Stigma (diamonds) and anther (dots) positions with respect to the perianth base for each flower. Individual flowers are arranged in order of increasing style length.

It is important to notice that anther position within the flowers followed a normal distribution for the whole population (Kolmogorov–Smirnov *d* = 0.075, *p* < 0.01, see dot distribution in Figure 2); it can be assumed that there was a (mean) anther position such that the probability of being the pollen source was maximal. This was important for the proxy for herkogamy we propose.

To identify trends underlying the observed inter-individual variation, we subjected the morphological data to a principal component analysis (PCA). The results showed a clear integration between most of the floral variables, which loaded heavily on the first axis. Only amplitude between stamens (difference between the highest and the lowest anthers for each flower) loaded significantly on the second axis (Table 2). The first two axes explained most of the variance of the data matrix (92.1%). Phenotypic integration for the population of Bertamiráns was 45%.

The flowers of *N. bulbocodium* produced 165,430 ± 25,400 pollen grains per flower, and none or very few were aborted (0.0–0.7%). Each flower contained a mean of 131 ± 27 ovules. Excluding the aborted pollen grains, flowers presented a pollen/ovule ratio (P/O) of 1309 ± 309 (Table 1).

Floral morphology and pollen loads on stigmas. Among the considered variables, only stigma exertion had a significant and negative effect on the female fitness variable (pollen load on stigmas)—the more loaded stigmas were those at the level of or within the corolla edge (Table 3).

Pollen found on the stigmas was mostly conspecific. Allospecific pollen was more abundant in only 12 out of 90 examined flowers (13%). For the remaining cases, the mean proportion of allo-/conspecific pollen was 0.1, and the median and mode were even lower (0.05 and 0, respectively). Therefore, most flowers received conspecific pollen only, and even those with allospecific pollen received more conspecific pollen. There was no correlation between conspecific and allospecific pollen loads (Kendall’s tau = 0.18 for conspecific to allospecific—entomophilous, and Kendall’s tau = 0.12 for conspecific to allospecific—anemophilous; *p* > 0.05 for both correlations).

### 2.2. Mating System

**Figure 3:**
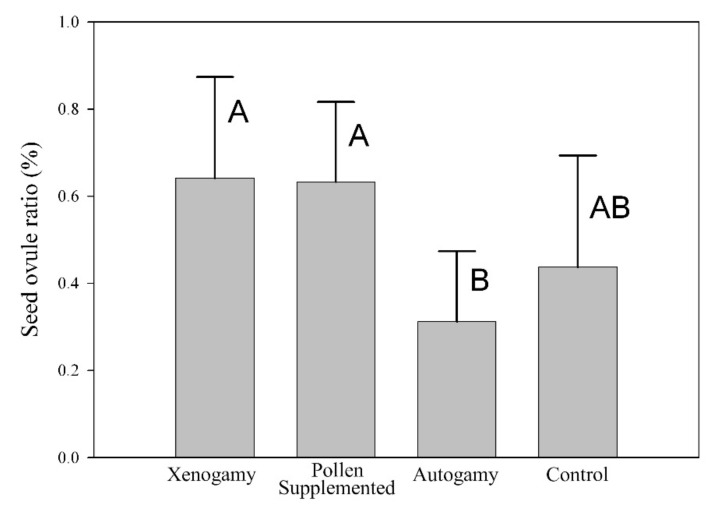
Results of the mating systems treatments carried out in *Narcissus bulbocodium*, expressed as mean ± SD of the seed/ovule ratio. Different letters reflect differences in the post hoc Tukey analysis.

The results of the hand pollination experiments are represented in Figure 3. Pollen source significantly affected the seed/ovule ratio (F_(3,43)_ = 5.07, *p* = 0.004). Xenogamous and supplementary pollination treatments produced the highest values (64.2% and 63.3%, respectively). A significantly higher number of seeds were produced after outcrossing compared to selfing; the estimated fitness of the latter was approximately half that of outcrossing (Figure 3). Taking into account the index defined by Jules and Rathcke [19], there was also a pollen limitation of 30.8%.

### 2.3. Self-Interference and Herkogamy

Our results showed deposition of self-pollen on the stigmas of bagged flowers in a variable number but usually few grains per stigma (range 0–453, median (quartiles) = 36 (4–216)). We found a negative correlation between the amount of self-pollen on the stigma and herkogamy—the more herkogamous flowers received fewer self-pollen on the stigma without the intervention of pollinators (Kendall’s tau = −0.43, *p* < 0.001). Emasculated flowers showed no pollen grains on their stigmas, proving that the bagging system was effective.

### 2.4. Pollinator’s Assemblage

**Figure 4:**
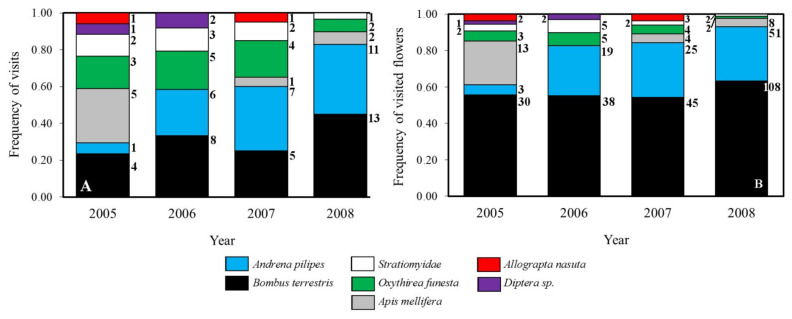
Frequencies of visits by each of the main groups of visitors of *Narcissus bulbocodium* (**A**) and percentage of visited flowers per visiting group over the total number of flowers observed (**B**); numbers are raw data. Because the spectra of floral visitors between the two studied populations were very similar, data from both sites were pooled in this figure. Most pollinators belong to Order Hymenoptera (*Andrena*, *Bombus*, *Apis*), followed by Diptera (*Allographta*, Stratiomydae, Diptera sp.) and Coleoptera (*Oxythyrea*).

The same seven species of insect visitors were observed pollinating the flowers of *N. bulbocodium* in both populations (Figure 4). Pollinators are generalists, with slight but not significant variation in the number of visits between years (GLM estimate = 0.16, *p* = 0.098), and no difference between populations (GLM estimate = −0.02, *p* = 0.917). The main visitor over the four years was *Bombus terrestris* followed by *Andrena pilipes*. In all cases it was observed that floral visitors came into contact with the anthers. Both populations presented very low visitation rates, below 0.8 visits per 15 min period. It is remarkable the among-year variability of *Apis mellifera* in both populations; although we have not studied this, several factors could account for that variability, as temperature variation, variability of their recruitment system of the species, and the somehow random beekeeping in northwest Spain with many small farms installing beehives occasionally as a secondary economic activity.

### 2.5. Pollen Dispersal Range and Precision

**Figure 5:**
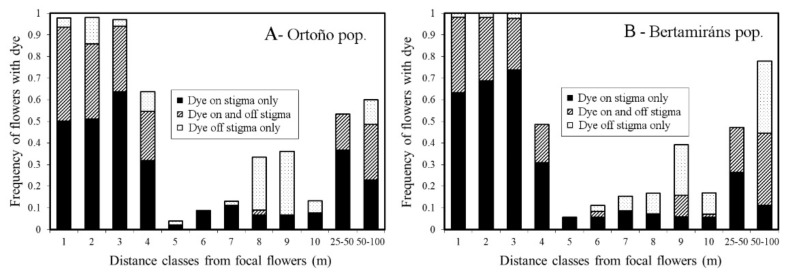
Cumulative frequencies of flowers with pollen substitute (dye) in relation to the distance classes from the focal flowers in the populations of Ortoño and Bertamiráns. The 10 first classes comprise 1 m each; the following from 25–50 and 50–100, respectively. For each class, the frequencies of flowers with (1) fluorescent dye on the stigmas only, (2) dye on and off the stigmas, and (3) dye off the stigmas only are shown.

Pollen dispersal range. The pollination range of *N. bulbocodium* was studied with dyed powder as a pollen substitute (Figure 5). Flowers with powder were unevenly distributed relative to focal flowers (for Ortoño flowers with pollen only on the stigma, Kruskal–Wallis H = 117.88, *p* < 0.01; with pollen only off the stigma H = 56.01, *p* < 0.01; with pollen both on and off the stigma H = 109.95, *p* < 0.01; for Bertamiráns flowers with pollen only on the stigma, H = 203.85, *p* < 0.01; with pollen only off the stigma H = 52.14, *p* < 0.01; with pollen both on and off the stigma H = 85.91, *p* < 0.01). These findings suggest that there was a significant link between the distance categories and the presence of dye on stigmas.

For both populations, most flowers (close to 100%) within 3 m from the focal flowers were visited by pollinators carrying powdered dyes, and about 50% in the fourth meter. From 4 m on, the frequency of pollinated flowers decreased significantly in both populations to a minimum at around 8–9 m from the focal flowers. From here on, pollen placed off the stigma increased up to between two (Bertamiráns) and four (Ortoño) times the number of successful placements. One remarkable and apparently contradictory fact is that long-distance pollination (from 25 to 100 m) was relatively successful, with approximately 50% of the total sampled flowers for both populations being pollinated at that distance. Furthermore, pollen in those cases was mainly “successful” since dye was mainly placed on the stigma or stigma and style.

**Figure 6:**
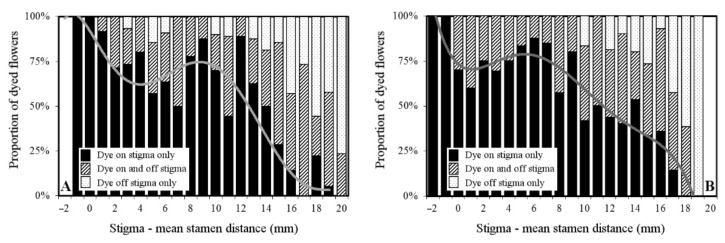
Proportion of the dyed flowers with dye powder on the stigma, off the stigma (on the style), and both on and off the stigma, relative to the proxy for herkogamy in the Ortoño (**A**) and Bertamiráns (**B**) populations of *N. bulbocodium*. Graphics include the fitting polynomial line for the “flowers with powder on stigma only”; according to the results of the cross-validation analysis, the best polynomial fitting for population (A) was degree of six, and for population (B) was a degree of five (see Section 4).

Pollen dispersal precision. Considering only the “dye-pollinated” flowers, the relationship between the proxy for herkogamy (distance from each individual stigma to the mean stamen height of the population) and pollination precision (as percentage of flowers with pollen substitute only on the stigma) was calculated. It can be seen in Figure 6 that as the proxy of herkogamy increased, the probability of getting fluorescent dye on the stigmas decreased. Nonetheless, the cross-validation resampling method had shown that the best-fitting models were sixth and fifth degree fitting curves for populations Ortoño (*R^2^* = 0.41) and Bertamiráns (*R^2^* = 0.30), respectively. Both curves showed a similar pattern with a maximum when herkogamy was close to zero, but with a secondary maximum at 8–12 mm for Ortoño and 5–9 mm for Bertamiráns. These latter values were close to the mean population herkogamy values (5.25 mm, see Table 1).

Precision decreased when the mean herkogamy distance increased beyond 12 mm at the Ortoño population and 9 mm at the Bertamiráns population. The proportion of “misses” (pollen substitute off the stigma) increased consistently, with a maximum at 19–20 mm in both populations.

## 3. Discussion

### 3.1. Floral Morphology and Integration

According to the inferences by Armbruster et al. [5] from the “correlation pleiades” of Berg, a higher degree of correlation should be expected between those floral characters directly influencing fitness than between vegetative traits. Phenotypic floral integration arises as a consequence of the stabilizing selection acting to enhance an optimal balance between male and female functions in animal-pollinated flowers [6], despite the variation of the balance between male/female functions among individuals and seasons (e.g., [20]); those populations with a higher dependence on their pollinators for reproduction will show more “integrated” flower phenotypes [5]. The high degree of floral integration in *N. bulbocodium* suggests this species is highly reliant on their pollinators. Although we have not analyzed the degree of integration of vegetative traits (not directly related to fitness), the population analyzed in this study showed high values of floral integration (45%) compared with other studies. For example, Ordano et al. [21] found an average of 21.5% in 36 species, and Ferrero et al. [22] found “high values” between 33.9 and 64.7%. High integration values increased the probability for a population to stayed close to the “adaptive ridge of highest fitness” as per the work of Armbruster et al. [13], but must be considered with caution because among-population variability can also be high [23].

### 3.2. Integration, Pollination, and Pollination Range

Armbruster et al. [5], as Berg [4] before them, suggested that limited variation in the floral characters should be expected in species highly dependent on pollinators; they found mean CV values between 7% for floral traits of species with “specific pollination systems” (i.e., specialized pollinators) and 18% for species with generalized or wind-pollinated systems. Our data on the variation of floral traits in *N. bulbocodium* showed intermediate variation, but closer to those described for specific pollination systems (Table 1). This is consistent with the assemblage of pollinators found (Figure 3); although mainly generalists, pollinators show substantial fidelity to this plant since pollen from other species was rarely found on its stigmas. Being a winter species, the scarcity of other plants in bloom is also contributing to this. Pollinators’ assemblage and fidelity can help to understand our results on the precision of pollen deposition. The pollinators of *N. bulbocodium* are efficient at distances above 25 m (Figure 5), which is coincident with the foraging pattern of the main pollinator, *Bombus terrestris*. It also has the largest body size of all pollinators recorded (widely around 15 mm [personal observation]; foragers likely larger than non-forager workers [24]), being well suited to pollinate the large flowers of *N. bulbocodium*. Although they can fly long distances, if there are flowers nearby, most *B. terrestris* workers feed within 100 m around the nest [25]. The foraging pattern of *B. terrestris* could probably also explain why pollination is more frequent at long than intermediate distances, while selfing and the action of less active pollinators as *Oxythyrea funesta* could explain the pollination at short range. Two results were found in this work that are linked to the behavior of pollinators: (1) visiting sequences of conspecifics at both short and long distances and (2) relatively high precision in the deposition of pollen on the stigmatic surface, even in long-distance pollination, could account for the relatively narrow variance of floral characters, closer to those of a specialized than to a generalist pollination system.

### 3.3. Mating System and Pollen Limitation

Although according to P/O ratio and the results of the mating experiments (Figure 3) *N. bulbocodium* is a facultative xenogamous (sensu Cruden [26]), it must be kept in mind that (1) seed production after autogamy is sensibly lower than the control, indicating a possible selection against autogamy, and (2) our fitness estimate is seed production, and therefore it is quite possible that some later-acting deleterious factors linked to inbreeding may be acting on germination, seedling, or later developmental phases. In such cases, the dependence on pollinators and outcrossing would be even greater, which could explain the similarity of this system with a “specific pollination system” as per Armbruster et al. [5]. Mixed mating systems and autonomous selfing can provide a reproductive assurance in environments where pollinators are either scarce or unpredictable or where the availability of outcrossing pollen is limited [15,27,28,29]. In the case of *N. bulbocodium*, a late-winter flowering species with a low floral visitation rate, autonomous selfing seems to be assuring pollination despite the pollen limitation in winter [30,31].

### 3.4. Herkogamy and Pollination

Among the morphological factors conditioning the female fitness proxy considered (pollen loads), it is remarkable that only the “exertion distance” (i.e., the distance between the stigma and the margin of the corolla) had a significant effect (Table 3). The negative nature of that relationship shows that those stigmas confined within the corolla have a higher probability of being pollinated; by including the sexual organs inside the cone-shaped corona, the flower facilitates the contact between those organs and flower visitors, promoting pollen deposition (self or outcross) on stigmas. This is important for the development of an “ordered herkogamy” (as per the work of Webb and Lloyd [12]), which is essential for the evolution of specialized herkogamy systems such as reciprocal herkogamy or heterostyly [12,22,32,33,34]. In the similar herkogamous species *Narcissus longispathus*, Medrano et al. [14] found that the highest rate of outcrossing occurred in flowers with stigmas within the corolla, in a position intermediate between the rim of the corolla and the anthers. These authors found a discernible trend such that flowers with intermediate values of herkogamy are at a relative outcrossing advantage over those having either smaller or larger anther–stigma separation. This led them to propose a scenario of stabilizing selection mediated by the pollinators for the maintenance of herkogamy in *N. longispathus*.

Several studies have shown in different plant families a positive correlation between outcrossing rates and herkogamy (e.g., *r* = 0.68 for *Mimulus ringens* L. [16], *r* = 0.71 for *Aquilegia caerulea* James [15]) and a negative correlation between herkogamy and selfing (*r* = –0.24 for *Aquilegia canadensis* L. [35]). As far as we are aware, there is only one exception to this monotonic trend—the above-mentioned findings in *Narcissus longispathus* [14], which seem to support the hypothesis that the main evolutionary force behind herkogamy is the promotion of outcrossing. However, Webb and Lloyd [12] suggest that the primary evolutionary explanation for herkogamy may be neither the promotion of outcrossing nor the reduction of self-fertilization, but the avoidance of self-interference because there are a number of instances of herkogamous self-incompatible species in which self-fertilization is already prevented (see also [36]). Our results seem to indicate that (1) outcross precision is maximal when the proxy of herkogamy is short or intermediate (Figure 6), and (2) herkogamy is negatively related to autonomous self-pollination, which results in lower reproduction (Figure 3). Therefore, the maximum precision at short herkogamy-proxy distances could be somehow penalized, while the intermediate could be both maximizing outcrossing and minimizing self-interference. In a species with a mixed mating system such as this, the most likely hypothesis is that both factors, promotion of outcrossing and avoidance of self-interference, are playing a role in the maintenance of herkogamy. In an optimal situation, the stigma should be distant from the own flower anthers and close to the average anther height of the population, but our results were not conclusive on this since the (actual) herkogamy value was not a significant factor on pollen load according to the multiple regression analysis results.

Whether it arises to promote cross-pollination or to avoid self-interference, the adaptive meaning of herkogamy seems to be unquestioned. According to the interpretation of Armbruster et al. [5] of the “correlation pleiades” hypothesis of Berg [4], one should expect a narrow variation for this character. However, we found high values of variation (CV = 32.2%), close to those of vegetative traits, while the mean variation of floral characters was between 6 and 18% (see [5]). This is not a peculiarity of our data, but instead appears to be a common characteristic of herkogamy (e.g., CV = 35% for *Aquilegia caerulea* [15], 34–40% for *Mimulus ringens* [16], and 23–43% for *Narcissus longispathus* [14]). This result stresses that herkogamy is not a simple character subject to the direct action of selection, but style and stamen length are the characters that selection acts on. As defined by Webb and Lloyd [12] as “a significant spatial separation of pollen and stigmas”, herkogamy has appeared many times in the evolution of flowering plants. Assuming a non-herkogamous ancestor, it takes only simple allometric variation in the length of either styles (as for *N. longispathus* [14]), or stamens (as for *Asarum* spp. [37]). Therefore, herkogamy should be considered a by-product of selection acting on style and/or stamen heights or the result of stochastic non-adaptive variation, bringing a universal adaptive origin of herkogamy into question. A paradigmatic case has been reported by Kelly [37], who has documented the origin of herkogamy in a self-pollinated group, resulting in a self-pollinated herkogamous species. No apparent adaptive interpretation can support the origin of herkogamy in such a case.

After answering the questions posed in Section 1, we could conclude that whatever the origin, our results seem to support that there is an optimal herkogamy distance such that outcrossing is maximized and self-interference is minimized, in accordance with the hypothesis of stabilizing selection in action. Although herkogamy possesses an apparent adaptive value, its high variability and several inexplicable cases as in *Asarum* L. [37] make the adaptationist explanation for the origin of herkogamy far from universal. Further studies should assess the effects of self-pollination in later stages of the life of the progeny. Several interesting questions regarding herkogamy must be addressed to develop a complete perspective of its adaptive significance, particularly concerning its ontogenetic origin in different lines of flowering plants.

## 4. Materials and Methods

All sampling and experiments were carried out in February 2005, and censuses of pollinators were conducted from 2005 to 2008.

### 4.1. Floral Morphology and Female Fitness

Floral characters. Floral lifespan was studied by monitoring flowers exposed to natural pollination; in the population of Bertamiráns, 25 floral buds were marked, and after anthesis, flowers were monitored daily until senescence. The receptivity of stigmas was examined each day using Peroxtesmo esterase indicator paper [38,39]. To study the morphometrics, we randomly collected 90 flowers from the same population and preserved them in 70% ethanol until analysis. In the laboratory, the following measurements were taken: (1) length of the corolla tube; (2) length of the ovary; (3) distance to stigma from the base of the corolla, avoiding the modification of the style curvature; and (4) distance to anthers from the base of the corolla, avoiding the modification of the filament curvature (Figure 1). Measurements were taken from photographs of each flower, first closed then dissected, using the AnalySIS 5.0 software package. Other indirect measurements were calculated, namely, herkogamy (distance between anther of the longest stamen and the stigma for each flower) and stigma exertion (distance between the stigma and the corona rim). Although some floral characters in some species are subject to ontogenetic change during the flower lifespan [12], 2 species of the genus, *N. longispathus* Pugsley and *Narcissus cyclamineus* DC., do not experience changes in herkogamy distance after anthesis ([14,40,41]; see [42] for a recent phylogenetic reconstruction of the genus). Thus, we can soundly assume that the observed variation reflects differences among individual plants rather than heterogeneity due to flower age. Even so, in order to minimize any possible effect of age-related variation in morphometric measures, we collected flowers approximately 3 days after anthesis since 3-day pistils and stamens are completely developed and receptive stigmas are clearly noticed from the naked eye.

To characterize the reproductive system of this species, we determined the pollen/ovule ratio (P/O) [26,43]. As it is likely that the number of viable pollen grains varies substantially among the anthers in a single flower, the total number of pollen grains was estimated in all anthers of 10 flowers (*n* = 10 plants) from the Bertamiráns population according to the procedure described by Castro et al. [44]. Each anther was placed on a microscope slide and stained blue with lactophenol to analyze pollen viability; then, a drop of 50% glycerin was added, and the preparation was compressed beneath a coverslip. The viable and non-viable pollen grains were counted under a light microscope (100× magnification). The number of ovules was counted for the same 10 flowers. Aborted pollen grains were excluded from the P/O determination.

### 4.2. Pollen Loads on Stigmas

Pollen grain loads on stigmas, a measure of female fitness, were counted in the same 90 flowers measured in the morphometric analysis. To this end, pistils were treated with sodium hydroxide (8 N) for 4 h, stained overnight with 0.05% aniline blue, and compressed in a drop of 50% glycerine (following [45]). Finally, they were observed through an epifluorescence microscope with a UV-2A filter cube. The following countings were recorded: (a) number of conspecific pollen grains on the stigma, (b) number of allospecific pollen grains on the stigma, and (c) number of pollen tubes growing through the stigmatic papillae and style.

### 4.3. Mating System

To determine the mating system, we investigated the effects of insect exclusion and pollen source (self versus outcross pollen) on seed/ovule ratio in the Bertamiráns population. The following pollination treatments were applied: (1) xenogamy: floral buds were emasculated and bagged until dehiscence, then hand-pollinated with a mixture of fresh pollen collected from 10 distinct plants and bagged again until withered (*n* = 11 flowers); (2) supplementary pollination: flowers were hand-pollinated with outcross pollen without bagging (*n* = 14 flowers); (3) obligate autogamy: floral buds were bagged until dehiscence, then hand-pollinated with their own pollen and bagged again until withered (*n* = 11 flowers); and (4) control: flowers without any treatment (*n* = 11 flowers). Since this species commonly produces a single flower per plant, a flower per individual was used in all cases. After 4–5 weeks, all fruits produced were collected shortly before dehiscence and examined under a dissecting microscope. Mature seeds and ovules that had failed to develop were counted for each fruit, and the seed/ovule ratio was calculated. This variable is henceforth considered the fitness indicator.

With the results obtained in hand pollination experiments, we also calculated the percentage of pollination limitation (PPL). This is a measure of the extent of reproductive success limitation by insufficient pollen delivery, calculated using the following formula:

PPL = 100 (PS − C) PS^−1^

where PS is the seed/ovule ratio of pollen-supplemented plants and C is the seed/ovule ratio of control plants [19].

### 4.4. Self-Interference and Herkogamy

To analyze possible spontaneous interference by self-pollen, we bagged a set of 30 floral buds from the Bertamiráns population without manipulation throughout the experiment. After 10 days, all flowers were transferred to the laboratory, and for each flower (a) stigma-highest anther distance (actual herkogamy) was measured, and (b) pollen load on stigmas was counted. Another 30 flowers were emasculated just before the opening of the stamens and bagged as a control to make sure that bagging was effective at preventing cross-pollination. All flowers were handled carefully to avoid any possible self-pollen contamination.

### 4.5. Pollinator’s Assemblage

To determine the floral visitor’s assemblage, we made direct observations of flower visitors in both populations over 4 years, from 2005 to 2008. The observations were performed on sunny days between mid-February to mid-March during the flowering peak, in several randomly selected areas of approximately 2 × 2 m where all the flowers (ranging from 24 to 32) could be easily observed. The observer was positioned approximately 1 m away from the study area and was able to monitor all floral visitors without disturbing their foraging behavior. At this distance, a skilled eye can identify small pollinators in this species. Visits were recorded during 15 min surveillance sessions at different hours of the day. A total of 112 censuses evenly distributed across the 2 populations and 4 years were performed, corresponding to 28 h of net observation (i.e., 14 censuses per population and year). Visiting species and total number of flowers visited per patch were recorded during each session. All floral visitors interacting with *N. bulbocodium* were recorded and further characterized as legitimate visitors if they contacted reproductive organs. The visitation rate of each population was assessed as the total number of visited flowers per population divided by the total number of surveillance sessions (after [46]).

### 4.6. Pollen Dispersal Range and Precision

To evaluate the pollen dispersal under natural conditions, we used fluorescent powdered dyes (Radiant Color, Richmond, CA, USA) as pollen analogues in the 2 studied populations (e.g., [47]). It has been previously observed that dye transfer closely resembles pollen transfer when bumblebees are the main pollen vector (e.g., [48,49,50]) despite the different dispersal properties of the dye and pollen grains [51]. Powdered dye was applied to the stamens of a set of 20 flowers in an area of approximately 1 m^2^ that acted as a focal source. Different powder colors were used for each population, and dye was re-applied every 3 days during the experiment. After 10 days, all flowers along 2 opposite linear 1 m wide transects starting in each pollen-source flower group were collected, and the distances from the source were recorded (distance classes: 1, 2, 3, 4, 5, 10, 25–50, 50–100 m; *n* ranged from 27 to 71 flowers per subgroup).

Flowers were preserved at −4 °C until analyzed. In the laboratory, flowers were examined under UV light using a stereo microscope to look for fluorescent powder. The distance between the stigma and the corolla basis was measured, and the presence/absence of powder on the stigma and/or style was recorded for each flower to evaluate the precision in pollen reception. The distance between the mean of the stamens of the population (pollen source) and each flower’s stigma (pollen target) was considered a proxy for herkogamy to analyze its relationship with the arrival of the pollen substitute.

### 4.7. Statistical Analyses

Floral morphology and female fitness. The relationships between the floral characters were analyzed by principal component analysis (PCA); since many variables tend to covary, the scores of the 2 first PCA axes were used as synthetic variables in the following analysis. A phenotypic integration index was estimated for the Bertamiráns population through the eigenvalues of a correlation matrix of floral traits that may condition pollination. The magnitude of phenotypic integration is represented by the integration index (the variance of the eigenvalues). Because sample size may vary among populations, the integration index was corrected by subtracting the expected value of integration under the assumption of random co-variation of traits (random integration = (number of characters − 1) number of plants^−1^). The integration index was expressed as a percentage of the maximum possible value, which is the number of traits considered (following [23]).

The influence of floral traits on pollen loads was tested with multiple regression. Since many of them tended to covary, we considered as independent variables the coordinates of each flower on the first 2 PCA axes from the previous section, and the derived variables herkogamy distance (style length minus the largest-stamen length) and stigma exertion (style length minus corolla length).

Mating system. The differences between mating treatments were evaluated by ANOVA and pairwise post hoc Tukey test, after arcsine transformation, which is recommended for proportion values as the seed to ovule ratio here. Normality was tested with the Kolmogorov–Smirnov test.

Self-interference and herkogamy. Self-interference and herkogamy were related with the Kendall rank-based measure of association test.

Pollinator’s assemblage. The variation in the number of visited flowers among populations and years was analyzed using a generalized linear model, with the dependent variable (number of flowers) adjusted to a Poisson distribution and a logarithmic link function for model responses.

Pollen dispersal range and precision. The frequencies of “pollinated flowers” (flowers with pollen substitute) between distance classes from a set of focal flowers were compared with a Kruskal–Wallis test.

To evaluate the influence of the proxy for herkogamy distance on the probability of obtaining pollen substitute only on the stigma as a measure of precision, we considered only the dye-pollinated flowers, and on them different fitting models were tested, selecting the optimal model by a k-fold cross-validation resampling method (*k* = 10). This method divides the n data (*n* = 258 and *n* = 271 for populations of Ortoño and Bertamiráns, respectively) into k groups, using k–1 groups to build the different models, while a group is left aside to evaluate the accuracy of the prediction of these models. This procedure was repeated for all groups, and thus each data point was left aside just once to determine the error of the individual prediction of the model. The selected model is that which minimizes the average of the quadratic errors of the predictions. Models tested were polynomials up to the eighth grade.

All analyses were performed with R [52], except for the cross-validation resampling method, which was developed in MATLAB.

Unless otherwise stated, all descriptive statistics in the text are presented as mean and standard deviation of the mean.

## Figures and Tables

**Table 1:** Descriptive statistics for the morphometric variables considered in *Narcissus bulbocodium* in the Bertamiráns population (Spain) (*n* = 90 plants for all variables except pollen and ovule number, and pollen/ovule ratio (P/O) ratio, *n* = 10; see Section 4).

	Ovary Length (mm)	Corolla Length (mm)	Style Length (mm)	Stamen Length (mm)	Stamen Amplitude (mm)	Herkogamy (mm)	Stigma Exertion (mm)	Pollen (*n*)	Ovule (*n*)	P/O
Min	4.59	27.15	28.77	18.53	1.43	1.69	−3.45	124398	97	832.9
Max	14.67	42.88	43.77	36.89	16.26	10.01	7.01	214202	169	1650.0
Mean	8.64	34.58	36.58	28.05	6.52	5.25	2.00	165089	131	1308.6
CV (%)	21.06	9.77	8.69	12.16	48.65	32.18	36.89	15.42	20.41	23.64

**Table 2:** Correlation between the floral variables and the three first axes (variable loads on the axis) of the principal component analysis for *Narcissus bulbocodium*.

Variable		Axis	
	I	II	III
Corolla length	0.90	−0.24	0.37
Style length	0.90	−0.34	−0.19
Stamen length	0.86	0.42	−0.20
Stamen amplitude	−0.16	−0.97	−0.11
% variance explained	59.79	32.35	5.58

**Table 3:** Results of multiple regression performed in *Narcissus bulbocodium* with number of conspecific pollen grains on stigmas (pollen load) as the dependent variable and stigma exertion distance, distance between stigma, and largest stamen (herkogamy distance) and the scores on the first two axes of the principal component analysis (PCA) as independent variables (*n* = 90). b: regression coefficients; β: standardized regression coefficients.

Variable	b	β	t	*p*
Constant	447.02		4.91	<0.001
Herkogamy distance	−23.64	−0.15	−1.39	0.17
Stigma exertion	−42.64	−0.33	−3.25	0.002
Scores on PCA1	7.59	0.05	0.45	0.65
Scores on PCA2	18.57	0.08	0.76	0.45

## Data Availability

The data presented in this study are available on request from the corresponding author.
